# Effectiveness of a behavioral lifestyle intervention on weight management and mobility improvement in older informal caregivers: a secondary data analysis

**DOI:** 10.1186/s12877-022-03315-w

**Published:** 2022-07-28

**Authors:** Xinran Liu, Jennifer King, Brandi Boak, Michelle E. Danielson, Robert M. Boudreau, Anne B. Newman, Elizabeth M. Venditti, Steven M. Albert

**Affiliations:** 1 Department of Behavioral and Community Health Sciences, University of Pittsburgh, 130 DeSoto Street, 6126 Public Health, Pittsburgh, PA 15213 USA; 2 Department of Epidemiology, University of Pittsburgh, Pittsburgh, PA USA; 3 Department of Psychiatry, University of Pittsburgh School of Medicine, Pittsburgh, PA USA

**Keywords:** Caregiver, Aged, Weight loss programs, Health promotion, Healthy lifestyle, Exercise, Community health workers, Community dwelling

## Abstract

**Background:**

Older informal caregivers are prone to sedentary behavior and obesity. With great caregiving burdens and frequent physical and mental distress, older informal caregivers may have low adherence and poor results in behavioral intervention for weight management. This study examined whether overweight or obese older informal caregivers could benefit from a behavioral weight management program as much as non-caregivers.

**Methods:**

The Mobility and Vitality Lifestyle Program (MOVE UP) was a pre-post, community-based, 13-month lifestyle intervention study to help older adults improve physical function performance and lose weight. We identified a subset of informal caregivers (*n* = 29) and non-caregivers (*n* = 65) from the MOVE UP participants retrospectively. Changes in lower extremity function, weight, depressive symptoms, and self-efficacy from baseline were compared between caregivers and non-caregivers using paired t-tests and ANCOVA.

**Results:**

Older informal caregivers had significantly lower session attendance rates than non-caregivers (67.7% vs 76.8%, *P* < 0.05), however, both informal caregivers and non-caregivers improved significantly in lower extremity function, weight loss, and self-efficacy in diet (*Ps* < 0.05). For each outcome, changes from baseline to the 13-month endpoint were the same among informal caregivers and non-caregivers.

**Conclusion:**

This study provides evidence that older informal caregivers can benefit from behavioral weight management interventions despite the challenge caregiving poses for effective self-care. Future behavioral intervention studies for older informal caregivers should adopt self-monitoring tools and extend the on-site delivery to home-based settings for higher adherence and greater flexibility.

**Trial registration:**

Registered at clinicaltrials.gov (NCT02657239).

## Background

In 2020, among 47.9 million informal caregivers (adults who provided unpaid regular care or assistance to a friend or family member who has a health problem or disability) in the United States, 19% were aged 65 years or older [1]. Compared to non-caregivers, older informal caregivers are more likely to have poor self-rated health, frequent physical and mental distress, and dissatisfaction with life [2]. Informal caregivers are also prone to sedentary behavior and obesity, especially those who are taking care of individuals with Alzheimer’s disease [3], possibly because of fewer opportunities to leave the home and other caregiving demands. Interventions focusing on weight management and reducing sedentary behavior could be important to help informal caregivers maintain a healthier lifestyle.

Physical activity (PA) and lifestyle intervention programs for middle-age to older caregivers have shown benefit for health outcomes. Several Randomized Controlled Trials (RCTs) conducted in 2002–2018 on a total of 677 caregivers reported that a PA intervention lasting from 2 to 12 months had positive effects on knowledge, readiness, and self-efficacy for PA [4, 5], with increases in PA levels, PA endurance, body strength and flexibility, and physical functioning, and with greater weight loss and muscle mass in intervention groups [5–11]. Pre-post PA intervention studies for caregivers also showed significant improvements in balance and number of exercise days [12, 13]. The interventions were mostly home-based [4, 5, 7, 8, 10] or group-based [6, 9, 11, 12, 14]. PA intervention methods include brisk walking [4, 9, 10], aerobic exercise [5–7], strength training [5–7, 9, 11, 12], tai-chi and yoga [12]. Multicomponent interventions also include healthy eating [9], psychoeducation [7], and group education [6]. However, most of the RCTs focused on caregivers of individuals with certain diseases (dementia [4, 5, 8, 13], Alzheimer’s disease [10], cognitive impairments [14], cancer survivors [6, 9, 11]), and were designed for dyads of caregivers and care recipients [9, 11, 13]. There is insufficient information about behavioral lifestyle interventions for a comprehensive group of informal caregivers in communities, with a gap in interventions that addressing overweight or obesity issues in this group. Therefore, the aim of this study is to examine if the effectiveness of a community-based behavioral lifestyle intervention for weight loss and mobility improvement is the same for older informal caregivers as for non-caregivers.

The Mobility and Vitality Lifestyle Program (MOVE UP) was a community-based lifestyle intervention program designed to help older adults improve physical function performance and lose weight [15]. Implemented in two waves in Pittsburgh area communities from January 2015 to June 2019, this 13-month multicomponent intervention has shown significant improvements in lower extremity function and weight loss in overweight or obese community-dwelling older adults [16]. In this ancillary study, we identified the caregiving status of MOVE UP participants and tested the representativeness by comparing their caregiving characteristics to a Pennsylvania representative sample. Then, we explored how older caregivers with overweight or obesity performed in this program relative to non-caregivers. Given that family caregivers usually spend hours taking care of recipients every day [17], and have poor mental health under great caregiving burdens [18], we hypothesize that caregiver participants may have lower adherence to the MOVE UP intervention, and are less likely to benefit as much from the MOVE UP intervention as non-caregivers.

## Methods

### Study design and intervention

The MOVE UP intervention was inspired by the following well-established health promotion programs: an evidence-based disease prevention program for older adults [19], weight loss interventions for overweight and obese individuals with diabetes [20–22], and other PA interventions targeting mobility disability among older people [23]. We applied a community-partnered approach by implementing the MOVE UP at community-based sites, and by recruiting and training community health workers (CHWs) for program delivery. The intervention process lasted for 13 months, and consisted of four phases: Phase 1 (month 1, 4 weekly sessions), introduction of screening and self-management for preventing late-life disease, disability, and functional decline [19]; Phase 2 (month 2–5, 16 weekly sessions), behavioral induction for healthy eating, PA, and weight loss; Phase 3 (month 6–9, 8 bi-weekly sessions) and Phase 4 (month 10–13, 4 monthly sessions) were reinforcing sessions focusing on self-management goals of healthy eating and PA. All 32 sessions were 1 h in length, lecture-based, held in groups, and led by CHWs on site. Based on the social-cognitive theory for health behavior change [24, 25], participants were encouraged to achieve and maintain a 7% weight loss goal from baseline and 175 minutes of weekly moderate intensity PA. The standard minimum goals were suggested by previous goal-based lifestyle interventions [20, 21], consistent with national public health recommendations and appropriate for older adults [26]. To help participants achieve and maintain these goals, CHWs instructed and assigned weekly Lifestyle Logs to participants for self-monitoring of daily calorie intake, home-based PA and weigh-ins.

The PA component was designed to help participants build their preferred home-based exercise program and reduce sedentary behaviors without supervision. To maximize behavioral adherence and minimize the risk of musculoskeletal injuries, participants were instructed to engage in planned moderate-intensity physical activity like brisk walking 5 days per week, beginning at 10 minutes per day, increasing progressively by no more than 5 min/day in 4-week intervals, and finally reaching at least 35 minutes per day. The in-class session introduced resistance training activities in Phase 2 (Session 19). To emphasize multi-modal physical activity training, materials from the Go4Life® “Workout to Go” [27] were provided, including pictures of older adults doing standing and seated strength, balance, and flexibility exercises. Participants were encouraged to take resistance training at least twice per week in addition to the 175 minutes aerobic activity goal, which is consistent with national public health recommendations for older adults [26]. Details of the intervention design [15] and main outcomes [16] were published previously.

This study was a secondary data analysis of the MOVE UP intervention study. After the completion of the parent MOVE UP study, from May 2019 to August 2019, we conducted a follow-up phone interview to identify participants’ caregiving status during the intervention and characteristics of their caregiving experience. Due to the availability of participants’ contact information and the accuracy of recall regarding caregiver status during the MOVE UP intervention, this secondary study only included participants in the second wave of MOVE UP implementation (December 2017 – June 2019).

The MOVE UP protocol and consent forms were approved by the University of Pittsburgh Institutional Review Board (IRB), and the study was registered at clinicaltrials.gov (NCT02657239). The study was conducted in accordance with the principles of the Declaration of Helsinki. All participants signed informed consents before enrollment in the MOVE UP study.

### Participants, interventionists and sites

Participants were recruited through multiple channels, including word of mouth, mass mailings, printed posters, newspaper advertisements and one feature story, and workplace announcements. Potential MOVE UP participants needed to pass a phone screen and a follow-up field screen for final enrollment. The inclusion criteria were the following: 60–75 years old, overweight or obese (Body Mass Index [BMI] 27–45 kg/m^2^), able to walk with or without an assistive device, and a medical clearance for participation from a personal physician. Exclusion criteria were the following: ongoing active cancer treatment (except nonmelanoma skin cancer), overnight hospitalization in the past 6 months, uncontrolled diabetes mellitus (fasting blood sugar > 300 and hemoglobin A1C > 11%), uncontrolled hypertension (systolic blood pressure > 180 or diastolic blood pressure > 110), history of bariatric surgery, and current use of weight loss medications. The research team also reviewed additional exclusionary factors that might impede participation, such as significant cognitive or psychiatric impairment, visual or hearing loss, or inability to read or communicate in English.

Potential community-based sites were recruited from prior studies or through the MOVE UP community advisory board, and needed to pass an on-site feasibility check for program delivery. CHWs were recruited from either community organization partners or from employees at the University of Pittsburgh as part of their regular employment. Training sessions (15 hours) for CHWs were carried out before the implementation of each of the first three phases, and included the knowledge for delivery, skills for group facilitation, and ethics for interventionists. The research team also supported CHWs during the MOVE UP implementation by optional monthly support calls and “Meet and Greet” events in communities.

### Data collection and measures

We conducted data collection in a pre-post study manner at each site: at recruitment (baseline) and the end of phase 2 (month 5), 3 (month 9) and 4 (month 13). We used data collected at months 9 and 13 as post-intervention data, depending on the availability of the latest assessment. Data covered the following six areas: *Demographic information* (baseline): age, gender, level of education, and race. *Body measurements* (all time points): weight, height, and BMI. *Medical History* (baseline) was measured by self-report [28]. *Lower extremity function* (all time points) was measured by the Short Physical Performance Battery (SPPB), a widely used and validated physical function measure that includes tests of gait speed (3 or 4 m walk test), standing balance, and chair-stand [29]. *Depressive symptoms* (all time points) were measured by the 20-item Center for Epidemiological Studies-Depression measure (CES-D) [30], a widely used scale in behavioral interventions with older adults. A cut-off point of 11 or higher indicates the diagnosis of mild depressive symptoms (MDS) [30]. *Self-efficacy* (all time points) in diet and exercise were measured by Weight Efficacy Lifestyle Questionnaire [31, 32], a theory-based measure used to assess participants’ confidence in their ability to follow the weight management program. Adherence was assessed by attendance rate of 32 sessions and number of Lifestyle Logs submitted (Maximum of 52 logs). Lower extremity function was the primary outcome; weight, depressive symptoms, self-efficacy in diet and exercise were secondary outcomes.

For the follow-up caregiver phone interview, we collected caregiving status and characteristics of the caregiving experience using the Behavioral Risk Factor Surveillance System (BRFSS) caregiver module [33]. Respondents were classified as caregivers if they responded yes to the following question in the phone interview: “People may provide regular care or assistance to a friend or family member who has a health problem or disability. During the time you participated in MOVE UP, did you provide regular care or assistance to a friend or family member who has/had a health problem or disability?” Additional caregiving characteristics collected were the following: care recipients’ relationship with caregiver, caregiving history, caregiving weekly work hours, care recipients’ main health problem, whether caregiver provided personal care and/or household tasks, and caregiving stress.

### Data analyses

Not all MOVE UP participants completed the follow-up caregiver telephone interview. For this reason, we first examined potential differences in baseline characteristics and adherence between MOVE UP participants who completed the phone interview versus those who were not, using t-test for continuous variables and Chi-squared tests for categorical variables. We then analyzed differences between caregivers and non-caregivers who participated in the follow-up caregiver study using the same statistical tests.

To test the representativeness of the MOVE UP caregiver participants, we also conducted a descriptive comparison using the results from BRFSS caregiver module, between our sample and a representative sample of community-dwelling older adults aged 60–75 years old, with overweight or obesity in Pennsylvania, from the BRFSS 2015 Data [34].

We used paired t-tests to evaluate changes in outcomes within caregivers and non-caregiver groups, respectively, and used ANCOVA with change-scores as the dependent variable to compare mean differences in outcomes between caregivers and non-caregivers over time (baseline vs. 5-months, baseline vs. post-intervention), controlling for demographic characteristics. We developed scatterplots with jittering to visualize changes in primary and secondary outcomes among caregivers and non-caregivers. Analyses were performed using R 4.0.3 and STATA 16.1 [35, 36].

## Results

For the MOVE UP intervention study, we enrolled a total of 303 participants, among whom 299 completed baseline assessments, and 240 finished the post-intervention outcome assessment. Twenty-two trained CHWs delivered the MOVE UP program in 26 community-based sites, with group sizes ranged from 6 to 15. Participants had a median attendance rate of 75.0% (Interquartile range [IQR], 53.1–87.5%) for 32 sessions, and submitted a median number of 19 Lifestyle Logs (IQR, 4–37 logs).

**Fig. 1:**
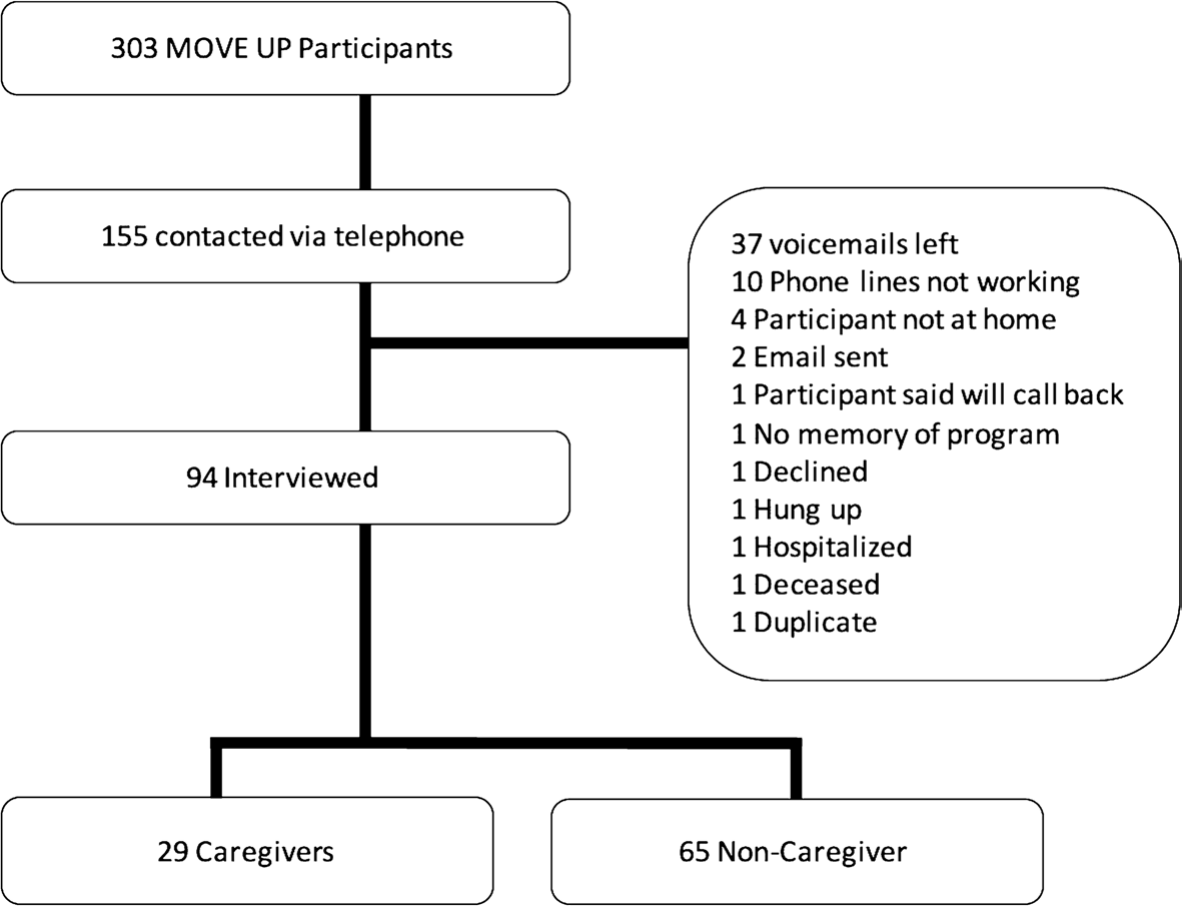
Derivation of MOVE UP Follow-up Caregiver Interview Sample

We reached out to 155 participants from the second wave of the MOVE UP implementation, and completed the phone interview with 94 (60.6%) of those reach, among whom 29 (30.9%) participants reported that they were providing regular care or assistance to adult recipients during the MOVE UP study. The flowchart for the follow-up caregiver phone interviews is depicted in Fig. 1. MOVE UP participants who completed the follow-up phone interview did not differ significantly from those who didn’t in demographic characteristics and baseline measures, except that interviewed participants had significantly higher adherence to the MOVE UP program (average attendance rate 74.0% vs 64.6%, *P* < 0.01; average number of Lifestyle Logs submitted 24.9 logs vs 19.7 logs, *P* < 0.05) (Table 1).

**Table 1:** Baseline Characteristics and participants’ adherence, grouped by interview and caregiver status

	Uninterviewed (***N*** = 205)	Interviewed (***N*** = 94)	***P***	Non-Caregiver (***N*** = 65)	Caregiver (***N*** = 29)	***P***
**Age, mean (SD)**	67.8 (4.2)	68.4 (4.0)	0.21	69.0 (4.2)	67.0 (3.3)	0.03
**Female, n (%)**	186 (91%)	79 (84%)	0.076	52 (80.0%)	27 (93.1%)	0.14
**Education, n (%)**			0.43			0.95
**Less than High School Diploma**	5 (2%)	3 (3%)		2 (3.0%)	1 (3.5%)	
**High School Graduate or GED**	42 (21%)	13 (14%)		10 (15.4%)	3 (10.3%)	
** Some College or Technical School**	54 (26%)	22 (23%)		14 (21.5%)	8 (27.6%)	
**Associate or Bachelor Degree**	63 (31%)	31 (33%)		22 (33.9%)	9 (31.0%)	
**Post-College or Professional Degree**	39 (19%)	25 (27%)		17 (26.2%)	8 (27.6%)	
**Race, n (%)**			0.10			1.00
**White**	128 (63%)	71 (76%)		49 (75.4%)	22 (75.9%)	
**Black**	66 (32%)	21 (22%)		14 (21.5%)	7 (24.1%)	
**Other race or two or more races**	9 (4%)	2 (2%)		2 (3.0%)	0 (0.0%)	
**BMI, mean (SD)**	34.8 (4.7)	34.9 (4.9)	0.83	34.9 (4.9)	35.1 (5.0)	0.80
**Weight, lb**	203.2 (32.2)	203.1 (34.3)	0.99	204.0 (33.5)	201.0 (36.5)	0.70
**Count, Medical Conditions, mean (SD)**	3.2 (1.7)	3.4 (1.8)	0.45	3.6 (1.9)	3.0 (1.7)	0.15
**Lower Extremity Function**						
**SPPB, mean (SD)**	10.5 (1.8)	10.5 (2.1)	0.76	10.5 (2.3)	10.7 (1.8)	0.63
**Gait speed, m/sec**	0.9 (0.2)	1.0 (0.2)	0.082	0.98 (0.2)	1.02 (0.2)	0.36
**Chair stands, sec**	11.5 (3.0)	10.8 (2.3)	0.082	10.9 (2.4)	10.7 (1.8)	0.67
**Mental Health**						
**CES-D, mean (SD)**	8.0 (6.6)	8.4 (8.0)	0.68	8.7 (8.1)	7.7 (8.0)	0.60
**MDS, n (%)**	58 (29%)	33 (35%)	0.34	24 (37.5%)	9 (31.0%)	0.54
**Self-efficacy**						
**Self-efficacy in Exercise, mean (SD)**	47.5 (21.1)	43.6 (20.8)	0.15	43.0 (21.1)	45.1 (20.2)	0.65
**Self-efficacy in Diet, mean (SD)**	55.7 (20.9)	53.4 (18.9)	0.35	52.0 (19.4)	56.4 (17.9)	0.30
**% of Session Attended, mean (SD)**	64.6 (2.1)	74.0 (2.0)	0.005	76.8 (2.0)	67.7 (4.7)	0.036
**# of Lifestyle Logs Submitted, mean (SD)**	19.7 (1.2)	24.9 (1.8)	0.015	26.5 (2.0)	21.3 (3.5)	0.17

Caregiver participants identified in the follow-up phone interview were mostly female (93.1%) and white (75.9%) (Table 1). Compared to non-caregivers, caregiver participants were significantly younger (mean age 67.0 vs 69.0, *P* < 0.05). Otherwise the two groups were similar in demographic characteristics, medical conditions, and baseline measures of weight, BMI, lower extremity function, mental health, self-efficacy in exercise and diet. Caregiver participants had a significant lower average attendance rate compared to non-caregivers (67.7% vs 76.8%, *P* < 0.05), and turned in 10% fewer weekly Lifestyle Logs out of 52 weeks, compared with non-caregivers (21.3 logs vs 26.5 logs), but this difference was not statistically significant.

### Caregiver status and characteristics

We identified 752 participants from the 2015 Pennsylvania BRFSS survey [35], who were community-dwelling, aged 60–75 years old, with overweight or obesity. Participants were weighted for representativeness, so only percentages and 95% confidence intervals (CI) were reported for comparing the caregiving status and characteristics of the caregiving experience with the MOVE UP sample (Table 2). 18.9% (95% CI: 15.6–22.9%) of our target older adults in Pennsylvania were caregivers, compared to 30.9% in the MOVE UP sample. MOVE UP caregiver participants were providing care mostly to parents (31.0%) or a spouse/partner (31.0%), whereas the percentages of these two categories were lower in the BRFSS caregiver subsample (21.2% [95% CI: 13.9–30.8%] for parents, 18.0% [95% CI: 11.4–27.1%] for a spouse/partner). Both MOVE UP caregiver sample and BRFSS caregiver subsample mostly had caregiving history longer than 6 months (MOVE UP 86.2%, BRFSS 72.5% [95%CI 62.0–81.0%]), and provided care or assistance less than 20 hours per week (MOVE UP 69.0%, BRFSS 66.9% [95%CI 56.2–76.1%]). The care recipients in the MOVE UP sample had a variety of health problems, most commonly dementia or other cognitive impairment disorders (20.7%), and arthritis/rheumatism (13.8%); compared to dementia or other cognitive impairment disorders (10.8, 95%CI 5.5–20.2%), heart disease or hypertension (10.6, 95%CI 5.5–19.6%), and cancer (9.0, 95%CI 4.7–16.6%) in the BRFSS caregiver subsample. During the MOVE UP implementation, 62.1% of the caregiver participants were providing personal care (45.6% [95%CI 35.2–56.5%] in BRFSS), and 75.9% of them were providing household tasks (72.1% [95%CI 60.3–81.5%] in BRFSS). Most MOVE UP caregiver participants reported that they felt stressed nearly always (27.6%) or quite frequently (20.7%).

**Table 2:** Comparison of MOVE UP caregiver participants and BRFSS caregiver subsample in Pennsylvania

	2015 Pennsylvania BRFSS subsample % (95% Confidence Interval) (*n* = 752)	MOVE UP sample N (%) (*n* = 94)
**1. Caregiver Status**		
Yes	18.9% (15.6–22.9%)	29 (30.9%)
No	80.4% (76.5–83.8%)	65 (69.2%)
Don’t Know/not sure	0.2% (0.0–1.6%)	0 (0.0%)
Caregiving recipients died in the past	0.3% (0.1–1.0%)	0 (0.0%)
Refused	0.1% (0.0–0.8%)	0 (0.0%)
**2. Care recipients’ relationship with Caregiver**		
Parent	21.2% (13.9–30.8%)	9 (31.0%)
Parent-in-law	7.2% (3.3–15.1%)	0 (0.0%)
Spouse/Partner	18.0% (11.4–27.1%)	9 (31.0%)
Sibling or sibling-in-law	12.6% (7.2–21.1%)	4 (13.8%)
Child	11.5% (5.1–23.8%)	1 (3.5%)
Grandparents	0.0% (0.0–0.0%)	0 (0.0%)
Grandchild	0.9% (0.1–6.3%)	0 (0.0%)
Other relative	12.3% (6.8–21.3%)	3 (10.3%)
Non-relative/family friend	16.4% (10.4–24.8%)	3 (10.3%)
**3. Caregiving history**		
< 30 days	13.0% (7.6–21.2%)	1 (3.5%)
1–6 months	14.6% (8.5–23.9%)	2 (6.9%)
6 months - 2 year	18.9% (12.0–28.6%)	8 (27.6%)
2–5 years	24.9% (16.1–36.3%)	7 (24.1%)
> 5 years	28.7% (19.6–39.9%)	10 (34.5%)
Don’t Know/ Not Sure	0.0% (0.0–0.0%)	1 (3.5%)
**4. Caregiving Work Hours**		
≤ 8 hrs/wk	60.2% (49.6–70.0%)	13 (44.8%)
9–19 hrs/wk	6.7% (3.5–12.4%)	7 (24.1%)
20–39 hrs/wk	7.5% (3.5–15.5%)	2 (6.9%)
≥ 40 hrs/wk	22.3% (14.5–32.7%)	6 (20.7%)
Don’t Know/ Not Sure	3.3% (1.0–10.0%)	1 (3.5%)
**5. Care recipients’ main health problem**		
Arthritis/Rheumatism	4.2% (2.2–7.8%)	4 (13.8%)
Asthma	0.6% (0.0–4.3%)	0 (0.0%)
Cancer	9.0% (4.7–16.6%)	3 (10.3%)
Chronic respiratory conditions	3.5% (1.5–8.1%)	3 (10.3%)
Dementia and other Cognitive Impairment Disorders	10.8% (5.5–20.2%)	6 (20.7%)
Developmental Disabilities such as Autism, Down’s Syndrome, and Spina Bifida	4.4% (1.5–12.1%)	0 (0.0%)
Diabetes	6.2% (1.7–20.3%)	3 (10.3%)
Heart Disease, Hypertension	10.6% (5.5–19.6%)	0 (0.0%)
Mental Illnesses	2.0% (0.5–7.6%)	1 (3.5%)
Other organ failure or diseases such as kidney or liver problem	1.2% (0.2–5.9%)	0 (0.0%)
Other	46.3% (35.8–57.2%)	7 (24.1%)
Don’t know/not sure	1.0% (0.1–6.9%)	1 (3.5%)
Refused	0.2% (0.0–1.6%)	1 (3.5%)
**6. Caregiver Provide Personal Care**		
Yes	45.6% (35.2–56.5%)	18 (62.1%)
No	54.4% (43.5–64.8%)	10 (34.5%)
Don’t know/Not sure	0.0% (0.0–0.0%)	1 (3.5%)
**7. Caregiver Provide Household Tasks**		
Yes	72.1% (60.3–81.5%)	22 (75.9%)
No	27.9% (18.5–39.7%)	6 (20.7%)
Don’t know/Not sure	0.0% (0.0–0.0%)	1 (3.5%)
**8. Frequency of Feeling Stressed**		
Never	–	5 (17.2%)
Rarely	–	5 (17.2%)
Sometimes	–	4 (13.8%)
Quite frequently	–	6 (20.7%)
Nearly always	–	8 (27.6%)
Don’t know/not sure	–	1 (3.5%)

### Outcomes

#### Lower extremity function

**Fig. 2:**
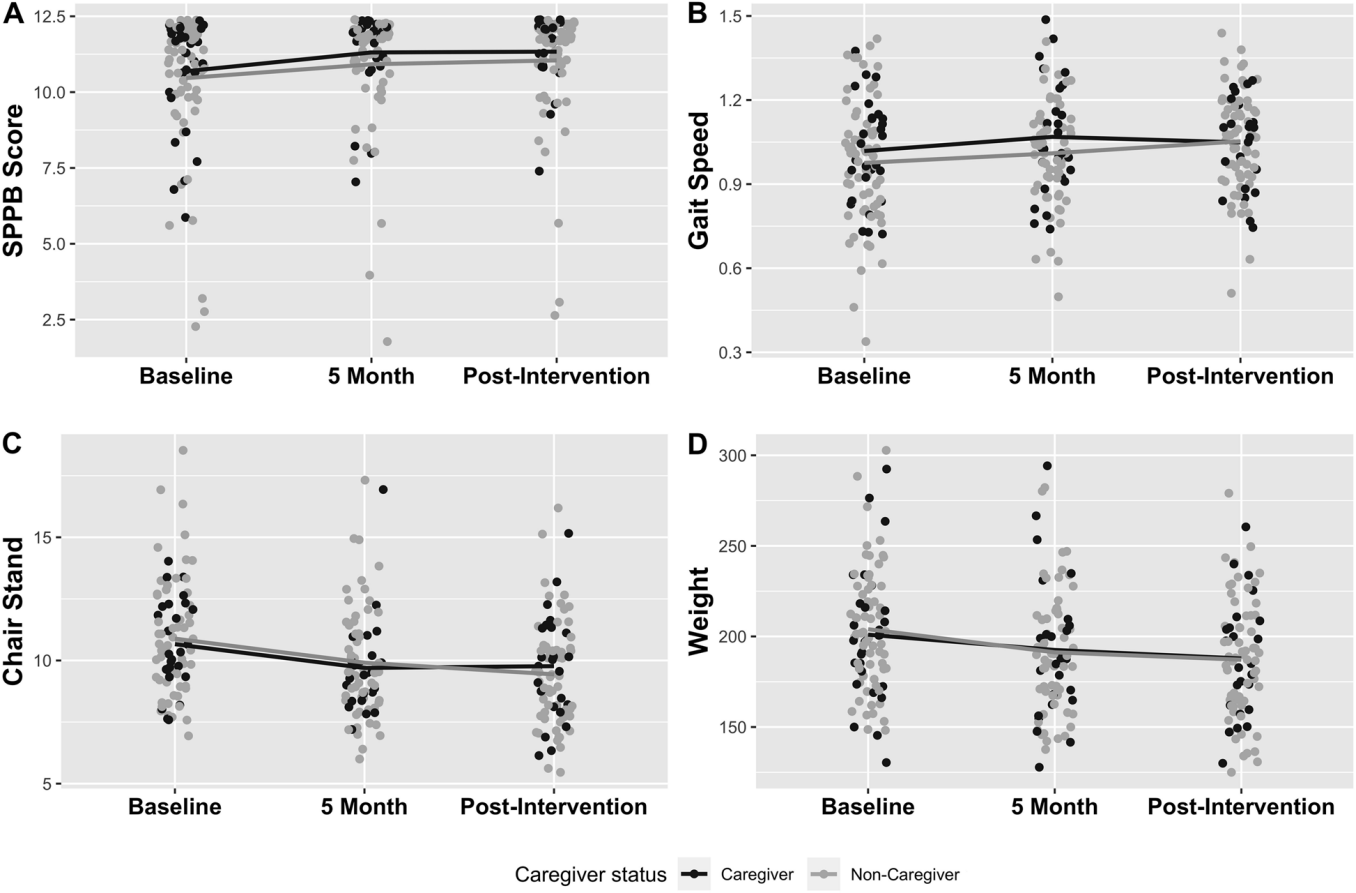
Change in SPPB Score, Gait Speed, Chair Stands, and Weight by Caregiver Status

Both non-caregiver and caregiver participants showed improvement in lower extremity function, evident in increasing SPPB scores (Table 3, Fig. 2-A). At baseline, 5 months, and post-intervention, the mean SPPB score of caregiver participants were 10.69, 11.31, and 11.33, respectively. For non-caregiver participants, the SPPB scores were 10.46, 10.92, and 11.05, respectively. Both groups had increased SPPB score significantly at month 5 (*P*s < 0.01), and maintained the increase until the post-intervention period (*P*s < 0.05). There were no significant differences in change of SPPB score from baseline between the two groups (*Ps* ≥ 0.05).

**Table 3:** Change in physical and mental outcomes by caregiving status

	Baseline	5 months	Post-Intervention
**Non-Caregiver**			
**SPPB, mean (SD)**	10.46 (2.26)	10.92 (1.94)^**^	11.05 (1.88)^**^
**Gait speed, m/sec**	0.98 (0.22)	1.01 (0.18)	1.05 (0.18)^**^
**Chair stands, sec**	10.89 (2.44)	9.91 (2.29)^**^	9.44 (2.16)^**^
**Weight, lb**	204.02 (33.48)	191.25 (32.60)^** a, b^	187.16 (32.66)^**^
**CES-D, mean (SD)**	8.67 (8.08)	7.45 (6.79)	8.05 (8.53)
**MDS, n (%)**	24 (37.50%)	17 (27.42%)	18 (27.69%)
**Self-efficacy in Exercise, mean (SD)**	42.98 (21.13)	47.31 (17.93)	47.51 (22.36)
**Self-efficacy in Diet, mean (SD)**	52.02 (19.36)	67.67 (17.75)^**^	65.20 (20.24)^**^
**Caregiver**			
**SPPB, mean (SD)**	10.69 (1.81)	11.31 (1.41)^**^	11.33 (1.20)^*^
**Gait speed, m/sec**	1.02 (0.18)	1.07 (0.20)^*^	1.05 (0.16)
**Chair stands, sec**	10.66 (1.81)	9.70 (1.95)^**^	9.77 (2.20)^**^
**Weight, lb**	201.00 (36.49)	192.53 (39.14)^** a, b^	187.84 (32.55)^**^
**CES-D, mean (SD)**	7.72 (8.00)	7.41 (6.25)	6.74 (8.03)
**MDS, n (%)**	9 (31.03%)	7 (25.93%)	4 (14.81%)
**Self-efficacy in Exercise, mean (SD)**	45.09 (20.22)	46.64 (18.98)	47.26 (21.43)
**Self-efficacy in Diet, mean (SD)**	56.42 (17.89)	66.23 (16.98)^*^	67.16 (19.23)^**^

* *P* < 0.05, compared to baseline

** *P* < 0.01, compared to baseline

^a^*P* < 0.05, change from baseline compared to the other group

^b^*P* < 0.05, change from baseline compared to the other group, age adjusted

Results of two sub-tests from the SPPB were also evaluated, gait speed (Fig. 2B) and time to complete five chair stands (Fig. 2C). At baseline, 5 months, and post-intervention, the mean gait speeds of caregivers were 1.02, 1.07, and 1.05 m/sec, respectively. The gait speed of caregivers increased significantly at 5 months (*P* < 0.05), but the increase did not maintain to post-intervention. For non-caregivers, the mean gait speeds at each timepoint were 0.98, 1.01, and 1.05 m/sec, respectively. The gait speed of non-caregivers was significantly improved at the post-intervention (*P* < 0.01). There were no significant differences in change of gait speed from baseline between two groups (*Ps* ≥ 0.05). Time to complete the five chair stands were 10.66, 9.70, and 9.77 sec for caregivers, and 10.89, 9.91, and 9.44 sec for non-caregivers. For both groups, the chair stands time at 5 months and post-intervention were significantly lower compared to baseline (baseline vs 5 months *Ps* < 0.01, baseline vs post-intervention *Ps* < 0.01). There were no significant differences in change of chair stands time from baseline between two groups (*Ps* ≥ 0.05).

#### Weight

Both non-caregiver and caregiver groups lost weight (Table 3, Fig. 2D). At baseline, 5 months, and post-intervention, the mean weights of caregivers were 201.00, 192.53, and 187.84 lbs., respectively. For non-caregivers, the mean weights were 204.03, 191.25, and 187.16 lbs., respectively. In both groups, weight at 5 months and post-intervention was significantly lower compared to baseline (baseline vs 5 months *Ps* < 0.01, baseline vs post-intervention *Ps* < 0.01). ANCOVA revealed a significant group by time interaction for weight at 5 months (*P* < 0.05), with the non-caregivers losing somewhat more weight during the period, and which remained statistically significant after adjusting for the age (*P* < 0.05). However, this difference was no longer evident at the post-intervention assessment (*P* ≥ 0.05).

#### Depressive symptoms

Non-caregiver and caregiver groups did not show significant changes or differences in CES-D score (Table 3). At baseline, 5 months, and post-intervention, the mean CES-D scores of caregivers were 7.72, 7.41, and 6.74, respectively. For non-caregivers, the mean scores were 8.67, 7.45, and 8.05, respectively.

#### Self-efficacy for exercise and diet

Self-efficacy in exercise did not differ between the two groups at each time point and did not show significant changes in either group (Table 3). However, for self-efficacy in diet, both groups showed significant changes across time. For both, self-efficacy in diet at 5 months increased significantly and maintained to post-intervention (*P*s < 0.05), but there were no significant differences between groups at any time point.

## Discussion

Although the sample size of MOVE UP caregiver participants was limited, caregivers identified using the BRFSS caregiver survey among the MOVE UP participants had similar caregiving experience (caregiving history, work intensity, and tasks) as those of the same age and BMI ranges, and community-dwelling status in the 2015 Pennsylvania BRFSS survey. Thus, results from this study may apply to older caregivers with overweight or obesity in Pennsylvania more generally.

Caregiver participants had significantly lower attendance rates in the MOVE UP program compared to non-caregivers, which may be due to their greater time pressure for caregiving tasks [17]. However, caregiver participants were able to complete and submit a large number of Lifestyle Logs similar to non-caregivers, which showed their engagement and fidelity to a behavioral lifestyle intervention at home. Previous findings from the MOVE UP implementation evaluation also demonstrated that it was the adherence to Lifestyle Log submission rather than the session attendance that had an independent significant improvement on participants’ weight loss. Future behavioral intervention studies targeting caregivers should continue to introduce Lifestyle Logs as an important self-monitoring tool, and extend the on-site delivery to home-based settings using an array of remote implementation tools, where family caregivers could have more flexibility and higher adherence.

Importantly, despite the stress and time demands faced by caregiver participants, they were as likely to benefit from the MOVE UP intervention as non-caregivers. In the primary outcome of lower extremity function as assessed by SPPB, both groups improved significantly and trajectories of change did not differ by caregiver groups. Similar findings were evident for chair stands, weight loss, and self-efficacy in diet. Caregiver participants lost less weight than non-caregivers at 5 months, but made up this difference in the post-intervention period.

To the best of our knowledge, this is the first study on behavioral lifestyle intervention for weight management to compare the effects among caregivers versus non-caregivers. The trends of increasing lower extremity function and decreasing weight among caregivers are comparable to previous RCTs [9, 11]. However, this research is limited by a retrospective design. Participants may not have had a clear recall of their caregiving status during the intervention. Also, not all participants were available through the phone interview, which could introduce selection bias. In addition, MOVE UP intervention materials did not focus on the topic of caregiving explicitly. It is possible that a behavioral weight management intervention geared to caregivers (e.g. by combining it with a caregiver training and support program) could offer greater benefit. Finally, caregiver burden depends to a large extent on the health status and degree of dependency of the care recipients [37]. This study was limited by the small sample size of older informal caregivers. Accordingly, we were unable to assess outcomes for important caregiver subgroups, such as those providing full vs. part time support, or personal care vs. support with household tasks.

## Conclusion

This study provides evidence that older caregivers can benefit from behavioral weight management interventions despite the serious challenges that caregiving poses for effective self-care. Future behavioral intervention studies targeting older caregivers should adopt self-monitoring tools and extend the on-site delivery to home-based settings for higher adherence and greater flexibility.

## Data Availability

The datasets used and/or analyzed during the current study are available from the corresponding author on reasonable request.

## Abbreviations

ANCOVA: Analysis of Covariance
BMI: Body Mass Index
BRFSS: Behavioral Risk Factor Surveillance System
CDC: Centers for Disease Control and Prevention
CES-D: Center for Epidemiological Studies-Depression
CHW: Community Health Worker
CI: Confidence Interval
IQR: Interquartile Range
IRB: Institutional Review Board
MDS: Mild Depressive Symptoms
MOVE UP: Mobility and Vitality Lifestyle Program
PA: Physical Activity
RCT: Randomized Controlled Trial
SD: Standard Deviation
SPPB: Short Physical Performance Battery
